# Obituary

**Published:** 1894-09

**Authors:** 


### OBITUARY.

### JOHN S. SAUNDERS.

Following is the report of a committee on resolutions appointed at a special meeting of the Massachusetts Veterinary Association, April 4th, 1894:

*Whereas*, Death has removed from among us our beloved member, Dr. John S. Saunders, and

*Whereas*, We feel *deeply* the loss sustained by us in the early demise of one whom we all have respected as a professional brother, and loved as a genial companion, be it

*Resolved*, That we hereby express our appreciation of his sterling qualities—honesty, charity, good fellowship, were the traits of his character. Devoted to his profession, an untiring worker, charitable to his colleagues, speaking ill of no man, his example is one for us all to emulate. By his death our association loses a valued member, the veterinary profession an ardent worker, the community an honest citizen. Be it

*Resolved*, That a copy of these resolutions be spread upon the records of our association, also forwarded to the family of the deceased, and to the veterinary journals for publication.

(Signed)
L. H. HOWARD,
AUSTIN PETERS,
JOS. H. STICKNEY,
F. H. OSGOOD,
H. P. ROGERS,
A. MARSHALL,
*Committee.*

